# The effects of a 25% discount on fruits and vegetables: results of a randomized trial in a three-dimensional web-based supermarket

**DOI:** 10.1186/1479-5868-9-11

**Published:** 2012-02-08

**Authors:** Wilma E Waterlander, Ingrid HM Steenhuis, Michiel R de Boer, Albertine J Schuit, Jacob C Seidell

**Affiliations:** 1 Department of Health Sciences and the EMGO Institute for Health and Care Research, Faculty of Earth and Life Sciences, VU University Amsterdam, De Boelelaan 1085, 1081 HV Amsterdam, The Netherlands

**Keywords:** Food prices, Pricing strategies, Randomized controlled trial, Pricing intervention, Fruits and vegetables, Virtual supermarket

## Abstract

**Background:**

Lowering the price of fruit and vegetables is a promising strategy in stimulating the purchase of those foods. However, the true effects of this strategy are not well studied and it is unclear how the money saved is spent. The aim of this study is to examine the effects of a 25% discount on fruits and vegetables on food purchases in a supermarket environment.

**Methods:**

A randomized controlled trial with two research conditions was conducted: a control condition with regular prices (n = 52) and an experimental condition with a 25% discount on fruits and vegetables (n = 63). The experiment was carried out using a three-dimensional web-based supermarket, which is a software application in the image of a real supermarket. Data were collected in 2010 in the Netherlands. Participants received a fixed budget and were asked to buy weekly household groceries at the web-based supermarket. Differences in fruit and vegetable purchases, differences in expenditures in other food categories and differences in total calories were analyzed using independent samples t-tests and multiple linear regression models accounting for potential effect modifiers and confounders.

**Results:**

The purchased amount of fruit plus vegetables was significantly higher in the experimental condition compared to the control condition (Δ984 g per household per week, *p *= .03) after appropriate adjustments. This corresponds to a 25% difference compared to the control group. Both groups had similar expenditures in unhealthier food categories, including desserts, soda, crisps, candy and chocolate. Furthermore, both groups purchased an equal number of food items and an equal amount of calories, indicating that participants in the discount condition did not spend the money they saved from the discounts on other foods than fruits and vegetables.

**Conclusion:**

A 25% discount on fruits and vegetables was effective in stimulating purchases of those products and did neither lead to higher expenditures in unhealthier food categories nor to higher total calories purchased. Future studies in real supermarkets need to confirm these findings.

## Background

In the search for effective interventions to stimulate healthier food choices, there is increasing recognition that the environment (either physical, social or economical) plays an important role in peoples' food choices, and is therefore potentially appropriate for interventions [1]. One of the potential successful interventions within this food environment are food pricing strategies [2].

Examples of potential pricing strategies include increased taxes on sugar sweetened beverages [3], snack foods [4], fatty or high-caloric foods [5-7]; or introducing healthy food subsidies [8]. In a previously conducted Delphi study [9], focus group study [10] and quantitative survey [11] expert and consumer viewpoints on the kind of pricing strategies that are considered to be most feasible and effective in stimulating healthy food choices were examined. All three studies investigated a wide range of strategies including taxes, subsidies, and insurance measures (e.g., receiving an insurance reduction when eating healthily). It was observed that experts and consumers agreed on the potential success of making healthy foods cheaper. Consumers indicated that they would eat more healthy foods if those products would become less expensive [10]. The experts judged subsidizing strategies, in addition to being effective, also to be feasible and affordable. Increased taxes were not viewed as being politically feasible [9]. A study by Herman et al. (2006) showed that subsidizing measures may indeed be effective. This study provided fruit and vegetable vouchers to low-income women and found that those were almost fully used in buying those products [12]. Neoclassical economic theory (Veblen, 1900) supports this finding by stating that consumers' choices are constrained by their available resources, and that the amount of purchases is a function of income, price and taste [13]. Therefore, lowering the price of healthier foods has good potential in raising sales of these products.

Still, prior to introduction, it is important to study the effectiveness of making healthy foods cheaper more extensively. It is important to consider both own price elasticity (e.g., the responsiveness of the quantity demanded of a certain good due to a price change of this good) and cross-price elasticity (e.g., the responsiveness of the demand for a good as a result of a price change of another good). The current evidence on the effectiveness of economic incentives in changing dietary behavior is limited and mostly restricted to small scale interventions [14] such as price intervention studies in high school cafeterias and vending machines [15,16]. To our knowledge, the only example of a randomized controlled trial studying the effects of pricing strategies on a larger scale is the New Zealand SHOP study. This study evaluated the effects of a 12.5% discount on healthier foods and nutrition education on supermarket purchases. The authors found that the price discounts alone raised the purchased number of healthy products [17].

Since SHOP is the only supermarket study on a healthy food subsidy yet, more research is needed to learn about its actual effects [18,19]. This study will therefore examine the effects of a 25% discount on fruits and vegetables in a web-based supermarket. Fruit and vegetables were chosen because they are generally viewed as being healthy and because the World Health Organization made a clear statement that the intake of those products should be promoted [20].

## Methods

### The three-dimensional web-based supermarket

**Figure 1:**
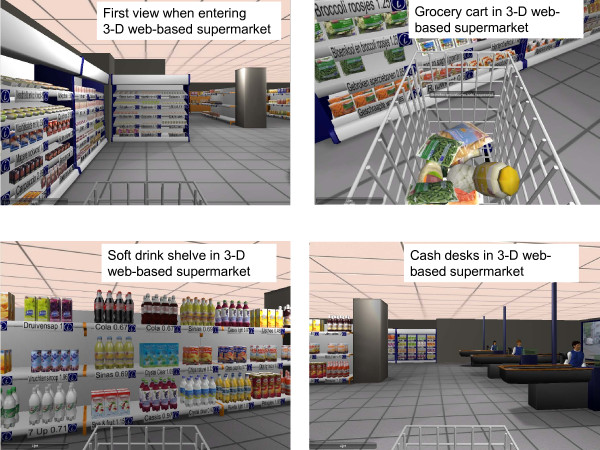
**Impression of the three-dimensional web-based supermarket**.

This study made use of an exclusively designed research tool which can be used to study pricing strategies in a supermarket environment without a complex implementation process: the Virtual Supermarket. The Virtual Supermarket is a three-dimensional (3-D) software application (Figure 1). A real life supermarket was used to design and to stock this web-based supermarket. The main features of the application are described below; additional information can be found elsewhere [21].

The 3-D supermarket application was designed in the image of a real supermarket using an Amsterdam branch of the Dutch market leader as a model. Photographs of real products were used to compose products for the software application and prices were made available through shelf labelling, meaning that a price tag was visibly present beneath each product type (comparable to a real supermarket). Food prices were based on the prices of the two Dutch market leaders, and the stock was also based on a real supermarket. For this purpose, figures provided by one of the major Dutch supermarket specialist journals (Distrifood) and information from the market leader's website were used [22]. An average Dutch supermarket offers about 7,000 different food products. Since this number contains for example around 200 different types of cheese and 250 different types of wine, it was decided to create a representative product selection using the 38 different food categories on the market leader supermarkets' website. These categories include, for example, potatoes, vegetables, pork, fish, soda, chocolate, and bread [22] (See Table 1 for an overview). Within each product category, a sample representing around 10% of the regular stock was selected by choosing popular and frequently consumed products. Due to a lack of sales data, the identification of popular products was conducted by the authors (WW and IS). This resulted in an assortment of 512 different food products. The actual total number of products was however larger because products could represent a number of product varieties. For example 'grapes' represented 'red and white grapes' and 'fruit yoghurt' represented 'peach/strawberry/and forest fruit flavours'. Further, to assure the availability of both healthy and unhealthy options, products meeting and not meeting healthy nutrition profiling criteria were chosen within each product category. The stock did not include specific brands.

**Table 1:** Outline of product categories and number of products in the web-based supermarket

	Food Category	Total products (n)	Healthy products (n)^a^
1	Potatoes and potato products	10	7

2	Fruits	10	10

3	Vegetables	41	41

4	Ready to eat meals	19	4

5	Meat/Fish/Poultry	29	13

6	Meat products	18	4

7	Salads (e.g., crab salad, egg salad, etc.)	8	3

8	Appetizers/snacks	6	1

9	Cheese	19	3

10	Dairy drinks (e.g., milk, yoghurt drink, etc.)	15	8

11	Desserts	21	4

12	(Whipped) cream	5	-

13	Butter	6	2

14	Eggs	2	-

15	Bread	15	6

16	Pastry	14	4

17	Snacks/refreshments	12	3

18	Frozen snacks	10	-

19	Ice (cream)	8	1

20	Frozen pastry	2	-

21	Coffee	7	-

22	Evaporated milk/sugar/sweeteners	9	2

23	Baking products	13	4

24	Sweet sandwich fillings	10	3

25	Breakfast products	13	6

26	Pasta/Rice/Noodles	12	4

27	Mixes for sauces	12	1

28	Seasonings	9	1

29	Herbs and spices	10	-

30	Oils/Sauces and pickles	26	9

31	Soups	12	2

32	Canned foods (excluding fruits and vegetables)	10	3

33	Beverages (excluding soda)	6	3

34	Soda	24	14

35	Alcoholic beverages	19	-

36	Candy	14	3

37	Chocolate	20	-

38	Crisps/nuts/toast	16	3

	**Total**	**512**	**172 (33.6%)**

^a^Healthy products are defined following the Choices front-of-pack nutrition label criteria which are based on the international WHO recommendations regarding saturated fat, trans fat, sodium, and added sugar [23].

Compared to previous studies using a supermarket model, such as Epsteins laboratory study where participants could choose between 30 healthier and 30 unhealthier products [24], the product assortment of the web-based supermarket is extensive and fairly represents a real supermarket stock. Also, compared to other web-based supermarkets using a drop down list from which participants could select their products [25] the shopping experience in our web-based supermarket more closely resembles a real shopping event.

### Study design

A randomized controlled study with two research conditions was carried out: 1) a control condition with regular prices; 2) an experimental condition with a 25% discount on fruits and vegetables. The discount level was chosen in congruence with previous studies [16,24]. Discounted products included fresh, frozen and canned fruits and vegetables. Fruit juices were not counted as fruits. Participants were randomly assigned to either the control or experimental group by using the Random Number Generator in Excel. The changed (discounted) prices were not made knowable to the participants in the discount groups. The prices appeared to both groups by neutral shelf price tags, without any further notion of the discounts. Moreover, participants were not aware of the research aims and were blinded with regard to assignment of the research conditions.

### Sample and recruitment

**Figure 2:**
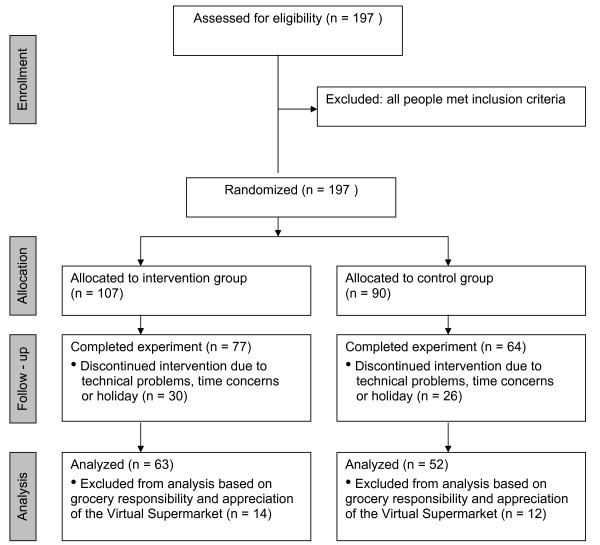
**CONSORT Statement Flow Diagram**.

A sample size was calculated using data on fruit and vegetable intakes (mean and standard deviations (SD)) from the Dutch National Food Consumption Survey 2003 [26]. In order to detect a significant difference of 400 g of total fruits and vegetables per person per week, a sample size of n = 104 was required. Participants were recruited through newspapers, the Amsterdam public library, and community centers in Amsterdam. Recruitment took place in 2010. Inclusion criteria were: being eighteen years of age or older, familiar with the Dutch language, and running an own household. N = 197 participants were randomized (See CONSORT Flow Diagram in Figure 2). The procedures followed in this study were in accordance with the ethical standards of the responsible institutional medical ethical committee. Study participants provided consent by emailing their approval for participation.

### Procedure

Most participants completed the experiment at home. Participants were instructed to undertake a typical shop for their household for one week. At the start of the program, participants were asked about their household composition and household income. Based on their answers, the program allocated a specific shopping budget. This amount was determined using data of the Dutch National Institute of Budget Education (NIBUD) and Statistics Netherlands (CBS). Following, participants found themselves with a grocery cart at the entrance of the three-dimensional supermarket. The shopping procedure was designed to be comparable to purchasing in real life. The application allows participants to walk along the shelves (using the cursor keys) and select products by a single mouse click. The selected product then appears in a shopping cart as well as on a list showing all groceries, their prices and total amount of money spent. This list could also be used to remove products. Furthermore, the program allows participants to read the nutritional label on the products by clicking on an information symbol next to the product. After finishing shopping, participants could go to the cash desk and, if the budget was not exceeded, they were directed to a closing questionnaire. Participants were not obliged to use the whole budget while shopping. After finishing the questionnaire all data were stored and send to our server automatically.

### Measures

The main outcome measure was fruit and vegetable purchases (in grams and items). Next, also purchased calories (kcal) and expenditures in unhealthier food categories were measured (e.g., desserts, soda, crisps, candy, and chocolate). Before entering the Virtual Supermarket, participants were asked some background variables including: sex; age; ethnicity; household composition; degree of being responsible for the groceries; weekly food budget; education level; employment status; and household income. Due to technical issues, age and income data were only available for about half of the sample. The program did collect complete income data when devising participants with their shopping budget, but did not store all these data adequately for further analysis. Finally, participants were asked to complete three questionnaires after shopping. The first questionnaire included a selection of questions from the seven "price perception construct scale items" by Lichtenstein et al. (1993) [27]. This questionnaire was included because consumers have very heterogeneous attention and reaction to prices [27]. The price perception scale items were specifically developed to capture such differences. For every construct at least two questions were included. The second questionnaire included the twelve item self-report index of habit strength [28]. Habit and impulsivity have been found to play a significant role in food choices, which could therefore moderate the effects of pricing strategies [29]. This questionnaire is validated to distinguish consumers with low or strong habits when grocery shopping. The final questionnaire included eight questions adding up to an appreciation score on the use of the Virtual Supermarket software. Questions included, for example, 'I could understand the program very well', and 'the products I purchased in the Virtual Supermarket are a fair representation of what I regularly buy in a supermarket'. This questionnaire was included to measure how well participants were able to use the web-based supermarket and to enable discrimination between participants with a high or a low level of understanding. Answers were measured on a 5-point Likert Scale. A final important measure was the assigned purchasing budget in the Virtual Supermarket which was calculated based on household size and standardized income. The assigned purchasing budget and scores on price perception, index of habit strength, and appreciation of the Virtual Supermarket were all dichotomized (0 = below mean; 1 = above mean) for further analyses.

### Statistics

First, all outcome measures were tested for an adequately normal distribution. Second, differences between the control and experimental group in fruit and vegetable purchases, purchased calories (kcal) and expenditures in the unhealthier food categories were tested using independent t-tests. Consequently, it was examined whether sex, assigned purchasing budget in the Virtual Supermarket, score on price perception, index of habit strength, or appreciation of the Virtual Supermarket modified the effect of the intervention on fruit and vegetable purchases. We focused on these variables because it can be expected that men and women or participants with a high versus low budget, high versus low score on habit, price perception or appreciation of the software react differently upon the price changes. For example, people who normally pay strong attention to food prices could be more susceptible to the price intervention. This analysis was done by using a multiple linear regression model with fruit and vegetable purchases as dependent variable, and research condition, the listed variables (dichotomized) and an interaction term as independent variables. Non-significant interaction terms were then removed from the model. For significant interaction terms it was planned to present the results separately for each group.

Third, analyses were conducted adjusting for potential confounders, including standard confounders (e.g., sex, educational level, and ethnicity), and confounders with a theoretically expected disturbing effect (e.g., the price perception score, index of habit strength, appreciation of the Virtual Supermarket, assigned purchasing budget in the Virtual Supermarket, responsibility for real life groceries, and household size). These confounders were included to adjust for differences in these variables between the control and experimental group. While no statistically significant differences were observed (Table 2) this adjustment was considered relevant due to our relatively small sample size. Household size was included as potential confounder because it can be expected that people with a larger household have other food purchases than people with a smaller household. Purchasing budget was included as an indicator for income (e.g., this variable was calculated based on household size and standardized income). The fully adjusted models were conducted separately for the different outcome measures. All analyses were conducted using SPSS statistical software (version 17.00, SPSS Inc, Chicago, IL). Finally, sensitivity analyses were performed to see whether adjustment for age and income, after imputation of missing values for these variables, would alter the associations under study. Using the multiple imputation procedure in STATA 11.2, we created 50 simulated databases in which missing values for age and income were imputed based on the regression of these variables with all the other relevant variables used in the main analyses. The final regression coefficients and corrected standard errors of interest were estimated based on these 50 imputed databases.

**Table 2:** Participant characteristics

		Control (n = 52)	Experiment (n = 63)	
			
		Mean (SD)	Mean (SD)	P(*t *- test)
		
Virtual shopping budget (€)		76.60 (39.53)	70.87 (31.27)	.40
Household size		2.63 (1.77)	2.43 (1.59)	.51

		N (%)	N (%)	P(Chi^2 ^test)
		
Sex (n = 115)	Female	43 (82.7)	48 (76.2)	.39

Age (n = 63) ^a^	18-31	3	2	.52
	32-46	8	15	
	47-61	16	14	
	62 +	2	3	

Ethnicity	Native Dutch	50 (96.2)	58 (92.0)	.46

Grocery responsibility	Totally responsible for groceries	37 (71.1)	33 (52.4)	.12
	Largely responsible for groceries	7 (13.5)	13 (20.6)	
	Partly responsible for groceries	8 (15.4)	17 (27.0)	

Education level	Low (primary/lower secondary)	20 (38.5)	5 (8.0)	< .001
	Medium (higher secondary/intermediate vocational)	22 (42.3)	46 (73.0)	
	High (higher vocational/University)	10 (19.2)	12 (19.0)	

Employment status	Employed	27 (51.9)	42 (66.7)	.27
	Other	25 (48.1)	21 (33.3)	

Household income (n = 63)	Low (0-2000)	11 (37.9)	9 (26.5)	.53
(gross monthly in €) ^a, b^	Medium (2000-3000)	8 (27.6)	9 (26.5)	
	High (3000+)	10 (34.5)	16 (47.0)	

Price perception score ^c^		40.7 (7.4)	43.1 (6.0)	.06

Habit score ^d^		35.5 (4.8)	37.2 (4.4)	.06

Appreciation score Virtual Supermarket ^e^		30.4 (4.2)	29.0 (3.7)	.06

^a^Due to technical issues, this question was not asked to all respondents

^b^The standard gross monthly income in the Netherlands (2010) is € 2,508 [30]

^c^Measured by 15 items (5-point Likert scale) from the seven "price perception construct scale items" (Lichenstein et al., 1993).

^d^Measured by twelve items (5-point Likert scale) self-report index of habit strength (Verplanken et al., 2003)

^e^Measured by eight items (5-point Likert scale) on the Virtual Supermarket software

## Results

### Participant characteristics

In total, n = 141 participants completed the study (non response = 28%). Because not all participants filled in their personal characteristics before randomization, it was impossible to compare the non responders with the final study sample. However, the final sample was of good quality and included participants within different socio economic positions (Table 2). From this sample, participants who stated being barely responsible for grocery shopping in real life and participants with a low appreciation score of the web-based supermarket were excluded from further analyses. A low appreciation score was set on approximately the bottom 10%, which included participants with a score of 22 or lower (score ranged from 16-40; mean = 28, SD = 5). In total, n = 26 participants were excluded (n = 12 from the control and n = 14 from the experimental group, Figure 2). Later, these participants were included in a sensitivity analysis.

The final study sample included n = 115 participants (n = 52 control and n = 63 experimental condition). Most participants were women (n = 91) and native Dutch (n = 108) (Table 2). There were no significant differences in participant characteristics between both groups, except for educational level (*p *< .001). Expenditures in the Virtual Supermarket were €60.98 (SD ± 27.33) in the control group and €58.86 (SD ± 24.15) in the experimental group (*p *= .66).

### Understanding and appreciation of the web-based supermarket application

First, was looked at the understanding and appreciation of the Virtual Supermarket. 91% of the participants scored ≥ 4 (scale 1-5) on comprehension of the software. Furthermore, 87% scored ≥ 4 on the question asking whether they could envision doing their normal groceries using the web-based supermarket. Finally, 80% scored ≥ 4 on the question asking whether their purchases at the web-based supermarket gave a good indication for their normal groceries.

### Differences in food purchases

#### Crude analyses

Overall, participants purchased 5,088 ± SD 2,392 g of fruits and vegetables for their household for a week (mean household size = 2.5 persons) (Table 3). Results showed that the experimental group purchased around 12.7% more fruit plus vegetables, but this was not statistically significant (*p *= .16). Further, it was found that the experimental and control group spent a comparable amount of money in other food categories (Δ€ -0.68, *p *= .89) and also purchased similar total calories (Δ-976 kcal, *p *= .78) (Table 3).

**Table 3:** Differences in food purchases and expenditures between the control and experimental group

	Control (n = 52)		Experiment (n = 63)			
	**Mean**	**SD**	**Mean**	**SD**	**Δ experimental - control**	**P** **(t-test)**
	
**FRUIT AND VEGETABLES**						
Fruit and vegetable expenditures (€)	11.49	4.74	9.71	4.88	-1.78 (15.5%)	0.05
Purchased vegetables (in items)	6.2	2.6	7.0	3.7	0.8 (12.5%)	0.20
Purchased vegetables (in grams)	2,879	1,241	3,191	1,675	311.7 (10.8%)	0.26
Purchased fruit (items)	1.7	1.1	1.9	1.4	0.2 (9.3%)	0.50
Purchased fruit (in gram)	1,877	1,082	2,171	1,599	293.4 (15.6%)	0.25
Total purchased fruit & vegetables (items)	7.9	3.1	8.8	4.4	0.9 (11.8%)	0.19
Total purchased fruit & vegetables (gram)	4,757	1,846	5,362	2,747	605.0 (12.7%)	0.16

**OTHER FOOD ITEMS**						
Expenditures in Virtual Supermarket (€)	60.98	27.33	58.86	24.15	-2.12 (-3.5%)	0.66
Total items purchased (n)	45.8	21.0	46.0	19.6	0.31 (.7%)	0.93
Total calories purchased (kcal)	36,343	20,379	35,367	17,543	-976 (2.7%)	0.78
Items in other food categories (n) ^a^	37.8	19.0	37.2	17.4	-0.60 (1.6%)	0.86
Expenditures in other food categories (€)^a^	50.42	24.58	49.74	22.41	-0.68 (1.3%)	0.40
Expenditures on desserts (e.g., pudding, yoghurt, etc.) (€)	1.60	1.50	1.62	1.62	0.02 (1.3%)	0.96
Expenditures on soda (€)	1.79	1.84	1.69	1.71	-0.10 (5.6%)	0.77
Expenditures on crisps (€)	0.93	1.06	0.71	1.00	-0.22 (23.7%)	0.26
Expenditures on candy (excl. chocolate)(€)	0.75	1.34	0.55	0.94	-0.20 (26.7%)	0.34
Expenditures on chocolate (€)	0.49	0.96	0.37	0.91	0.12 (24.9%)	0.49

^a^Total expenditures in food categories other than fruit and vegetables

#### Effect modification

Second, we studied whether some relevant variables modified the price intervention effects. None of the interaction terms was statistically significant. This indicates that the price discounts did not have a differential effect among men and women, on participants with a low versus high assigned budget or on participants with high versus low scores on price perception, habit, or appreciation of the web-based supermarket. The interaction terms were therefore removed from the model.

#### Corrected analyses

Finally, analyses were conducted adjusting for standard and theoretically expected confounders. Results showed that adjustment for these variables led to a statistically significant intervention effect of the 25% discount on the total amount of fruit and vegetables purchased (in gram) (B = 984; 95%CI: 97, 1,872; *p *= .03). The intervention group purchased around 25% more fruits and vegetables than the control group, which points to a price elasticity of 1.0^a^. Differences between both research conditions for single fruit (B = 481; 95% CI: -69, 1,030; *p *= .09) and single vegetable purchases (B = 504; 95%CI: -64, 1071; *p *= .08) were yet again large but remained not statistically significant (Table 4). Similar to the crude analyses, it was found that both groups had similar expenditures in unhealthier food categories and purchased similar total calories (kcal) (Table 4). The most important confounder in the model was the available shopping budget in the web-based supermarket. This variable was based on household composition and income, and revealed a strong positive association with the outcome measures.

**Table 4:** Intervention effect of the 25% discount on fruits and vegetables on food purchases and expenditures in the Virtual Supermarket

	B	Lower 95% CI	Upper 95% CI	P value
**FRUIT AND VEGETABLES**				
Fruit and vegetable expenditures (€)	-1.22	-3.04	0.60	0.19
Purchased vegetables (in items)	0.92	-0.36	2.19	0.16
Purchased vegetables (in grams)	504	-64	1,071	0.08
Purchased fruit (items)	0.41	-0.07	0.90	0.09
Purchased fruit (in gram)	481	-69	1,030	0.09
Total purchased fruit & vegetables (items)	1.33	-0.16	2.82	0.08
Total purchased fruit & vegetables (gram)	984	97	1,872	0.03*

**OTHER FOOD ITEMS**				
Expenditures in Virtual Supermarket (€)	0.97	-6.14	8.07	0.79
Total items purchased (n)	3.58	-2.22	9.38	0.22
Total calories purchased (kcal)	2,327	-3,494	8,147	0.43
Items in other food categories (n) ^a^	2.25	-2.91	7.41	0.39
Expenditures in other food categories (€) ^a^	2.19	-4.12	8.50	0.49
Expenditures on desserts (e.g., pudding, yoghurt, etc.) (€)	0.13	-0.45	0.72	0.65
Expenditures on soda (€)	-0.03	-0.73	0.67	0.93
Expenditures on crisps (€)	-0.27	-0.66	0.13	0.18
Expenditures on candy (excl. chocolate) (€)	0.07	-0.39	0.53	0.78
Expenditures on chocolate (€)	-0.22	-0.61	0.18	0.28

Linear regression model corrected for: sex, education level, ethnicity, responsibility for real groceries, price perception score, index of habit strength, appreciation of the Virtual Supermarket, household size and virtual shopping budget

*Significant at *p *< .05

#### Sensitivity analyses

Sensitivity analyses on the whole study sample (including participants with low scores on the Virtual Supermarket software and participants that were not responsible for groceries in real life) revealed similar results as the analyses on the sample excluding these participants. Furthermore, sensitivity analyses additionally adjusting for age and income (after imputation of missing values for these variables) revealed comparable results as the principal corrected analyses.

## Discussion

Results of this randomized controlled trial showed that a 25% discount on fruits and vegetables was significantly associated with higher total fruit and vegetable purchases in a web-based supermarket. The results showed that, after appropriate adjustments, the experimental group purchased 984 g more fruits and vegetables for their household for a week than the control group, which indicates a 25% difference. This difference points to a price elasticity (PED) of 1.0 and was independent on scores on habit and price perception. Also it was revealed that the discount on fruits and vegetables neither lead to higher expenditures in other (unhealthier) food categories nor to a higher total amount of calories purchased. These findings could have important implications for public health.

One rationale for introducing food pricing strategies is that monetary costs of a healthy diet may form an important barrier for low-income consumers in adopting such a diet [31]. Numerous studies have shown that nutrient-rich, low-energy-dense foods (e.g., fruits and vegetables) are generally relatively more expensive than high-energy-dense, fat and sugar rich foods [32-34]. In addition, it is suggested that in the current market, fruit and vegetables are promoted less than more profitable, highly processed foods containing more fats and sugars [35,36]. Since different studies have shown that, especially for low-income consumers, price is a major factor in food choice [37-39], pricing strategies are promising in stimulating healthier food alternatives. Already, marketing research has indicated price as a key tool in directing consumer behavior [40].

So far, the evidence on the effects of food pricing interventions was mostly restricted to interventions in smaller environments such as vending machines or work-site cafeterias. To our knowledge, our study is one of the first experimental studies on the effects of discounting fruits and vegetables in a virtual supermarket environment. When our results are judged against comparable studies, our findings are similar. First, The New Zealand SHOP study found that a 12.5% price reduction of healthier foods lead to 11% more healthy food purchases [17]. Also an economic modeling study by Jensen and Smed found that reducing VAT on fruits and vegetables from 25% to 12.5% lead to an increase in sales of 8% of those products [41]. Finally, French et al. conducted an experiment in high-school canteens and found that a 50% discount on fruits and baby carrots lead to a fourfold and twofold increase in sales respectively [16]. All together, there is increasing evidence that lowering the prices of fruits and vegetables is effective in stimulating the purchase of these foods. Recently, Andreyeva and colleagues published a review on the PED of food. Based on a selection of 160 studies, they concluded that food is elastic and that the highest PED was found for food away from home (restaurant meals and fast food), soft drinks, juice, meats, and fruit [19].

Nevertheless, there are also studies reporting possible negative side effects of subsiding healthier foods. For example, a study by Epstein and colleagues on a purchasing task in a laboratory setting found that discounting healthy foods with 12.5% or 25% lead to an increased number of total purchased calories since respondents did not only increase healthy, but also unhealthy food purchases [24]. A following relevant consideration regarding the effects of lowering fruit and vegetable prices is that people may purchase more of those products *additional *to their regular purchases instead of replacing other products by fruits and vegetables. In our study, we did not find that people spent the money they saved from the discounts in other (unhealthier) food categories. Also we found that both groups purchased similar amounts of calories and a similar number of products. An explanation for this difference in findings may be the studied product assortment. In Epsteins' study, people were able to choose between 30 healthier and 30 unhealthier products, whereas our web-based supermarket had a variety of 512 products. In addition, we only discounted fruits and vegetables whereas Epsteins' study discounted a wider range of healthier products [24]. This means that a fruit and vegetable subsidy may have better overall effects on food purchases than a discount on all healthier foods. Nevertheless, it is important to study this compensation effect carefully in experiments in real supermarkets, under different circumstances and by incorporating overall household expenditures (also outside the supermarket).

Another important aspect is that our results may be an underestimation because the discounts in the web-based supermarket were silent. Normally, when products are discounted, effort is made to draw people's attention by using signs or advertisements. Previous authors have suggested that people have a poor reflection of prices [42] and by using additional strategies; people become more aware of the discounts. Also, people have the tendency to buy a product simply because it is on sale [43,44].

The results of our study indicate that a discount on fruit and vegetables is effective in stimulating purchases of those products. Still, our study found only significant effects on fruit and vegetables *combined *and not for fruit or vegetable purchases separately. Nevertheless, the separate effects (+504 g vegetables and +481 g fruit per household per week) were also quite large and are considered relevant. These numbers point to a difference of 29 g and 28 g per person per day respectively. The latest Dutch Food Consumption Survey (2007-2010) showed that adults in the age 30 - 51 consumed a daily average of 121 g of vegetables and 77 g of fruit [45]. Increasing these numbers up to recommended levels of 200 g of fruit and vegetables per day could have large implications for public health [46]. An explanation for the non significant results, however, can be found in a lack of power. The used standard deviations in the power calculation were much smaller than the standard deviations found in our study. Therefore, a larger sample than expected was required to find significant results. It is therefore important to study the effects of fruit and vegetable price discounts in a larger sample. Such a study is also vital to gain more insight into the effects for specific groups, such as people with a low income or for ethnic minorities. Financial barriers against buying sufficient fruits and vegetables principally apply to low-income groups [10,11]. In our study, a majority of study participants had a standard income or above, making that their income was relatively high. Nevertheless, our results indicate that discounting fruits and vegetables was effective in this relatively high income sample as well, meaning that it can be expected that this strategy is equally (or even more) effective among people having limited financial recourses. Finally, results can not be directly generalised to populations with different eating habits and a different culture as opposed to the Netherlands (such as other EU countries or the US). Nevertheless, seen the generally low fruit and vegetable consumption in the entire EU [47] and also in the US [48] it can be expected that lower fruit and vegetable prices can have similar (or even greater) effects there as well.

A strong merit of our study is the use of the three-dimensional web-based supermarket which closely images a real shopping experience. Nevertheless, the assortment of the web-based supermarket is not as extensive as a real supermarket. Also, the Virtual Supermarket does not give insight into how people may shift to non-food items as a consequence of the price changes. Besides, the results are limited to a supermarket environment and do not give insight into effects at other point of purchase settings. Nevertheless, people buy most of their food at supermarkets (Dutch supermarkets' market share in 2011 was 86% [49]) and this seems thus the most obvious environment for interventions. Another limitation is that people may react differently in a real shopping situation with real products and real money compared to our web-based situation. Still, a large majority of the participants stated that their purchases in the web-based supermarket resembled their regular food purchases. Also, participants who had trouble in understanding the application were excluded from analysis. Furthermore, there is evidence that peoples' virtual behavior largely corresponds with their actual behavior. Sharpe et al. (2008) validated meal and beverage choices made in a virtual road trip survey by comparing those choices with choices made in a real McDonalds a week later. The authors found that peoples' simulated purchase behavior is highly predictive of their actual behavior [50]. Moreover, compared to previous studies where a supermarket environment was modeled using only 60 products [24] or using online drop-down lists [25], our three-dimensional, 512 products containing application seems a good quality research instrument. Unlike this, it is important to validate our results in a real shopping environment. A final limitation of our study is that some selection bias may have occurred because participants were self-selected. Still, participants were not aware of the research aims and were blinded with regard to assignment of the research conditions, which is considered a merit of our study.

## Conclusion

This study brings important new evidence into the effectiveness of reducing fruit and vegetable prices by a randomized controlled trial in a unique three-dimensional web-based supermarket. The results of this study revealed that a 25% discount on fruits and vegetables lead to substantial higher fruit and vegetable purchases (nearly 1 k gram per household per week) in the discount versus control group. Also, the study revealed that the discounts neither lead to higher expenditures in other food categories nor to higher calorie purchases. Future studies should expand these findings to a real supermarket setting. It is important that such studies focus on the effects on overall consumption along with the specific effects of pricing strategies among low-income consumers.

## Endnotes

^a^Price elasticity of demand (PED) refers to the responsiveness of the quantity demanded (ΔQd) of a good due to a price change (ΔP) of this good. Goods are seen as elastic if the PED > 1, using the following formula: $PED=\frac{\Delta Qd/Qd}{\Delta P/P}$[51].

## Competing interests

The authors declare that they have no competing interests.

## Authors' contributions

WEW was involved in the conception and design of the experiment, acquisition of the data, analysis and interpretation of the data, in drafting the manuscript and has given final approval of the version to be published. IHMS was involved in the conception and design of the experiment, in revising the manuscript for important intellectual content and has given final approval of the version to be published. MRdB (statistician) was involved in analysis and interpretation of the data, in revising the manuscript for important intellectual content and has given final approval of the version to be published. AJS was involved in the conception and design of the experiment, in revising the manuscript for important intellectual content and has given final approval of the version to be published. JCS was involved in the conception and design of the experiment, in revising the manuscript for important intellectual content and has given final approval of the version to be published.
